# Surface buildup dose dependence on photon field delivery technique for IMRT

**DOI:** 10.1120/jacmp.v5i2.1966

**Published:** 2004-08-16

**Authors:** Shigeru Yokoyama, Peter L. Roberson, Dale W. Litzenberg, Jean M. Moran, Benedick A. Fraass

**Affiliations:** ^1^ Department of Radiation Oncology University of Michigan Medical Center Ann Arbor Michigan 48109‐0010

**Keywords:** buildup dose, IMRT, high‐energy photon dosimetry

## Abstract

The more complex delivery techniques required for implementation of intensity‐modulated radiotherapy (IMRT) based on inverse planning optimization have changed the relationship between dose at depth and dose at buildup regions near the surface. Surface buildup dose is dependent on electron contamination primarily from the unblocked view of the flattening filter and secondarily from air and collimation systems. To evaluate the impact of beam segmentation on buildup dose, measurements were performed with $10\times 10\;{\text{cm}}^{2}$ fields, which were delivered with 3 static $3.5\times 10\;{\text{cm}}^{2}$ or $3\times 10\;{\text{cm}}^{2}$ strips, 5 static $2\times 10\;{\text{cm}}^{2}$ strips, 10 static $1\times 10\;{\text{cm}}^{2}$ strips, and $1.1\times 10\;{\text{cm}}^{2}$ dynamic delivery, compared with a $10\times 10\;{\text{cm}}^{2}$ open field. Measurements were performed in water and Solid Water using parallel plate chambers, a stereotactic diode, and thermoluminescent dosimeters (TLDs) for a 6 MV X‐ray beam. Depth doses at 2 mm depth (relative to dose at 10 cm depth) were lower by 6%, 7%, 11%, and 10% for the above field delivery techniques, respectively, compared to the open field. These differences are most influenced by differences in multileaf collimator (MLC) transmission contributing to the useful beam. An example IMRT field was also studied to assess variations due to delivery technique (static vs. dynamic) and intensity level. Buildup dose is weakly dependent on the multileaf delivery technique for efficient IMRT fields.

PACS numbers: 87.53.‐j, 87.53.Dq

## I. INTRODUCTION

Intensity‐modulated radiotherapy (IMRT)^(1)^ is a technical improvement in the delivery of radiotherapy requiring the use of optimized treatment planning for the specification of field positions and intensity patterns. Inverse planning optimization^(2)^ utilizes *a priori* knowledge of the desired location and magnitude of radiation dose. The optimization tools require an accurate calculation of delivered dose associated with each field and field‐intensity pattern. In some treatment sites (e.g., head and neck),^(^ ^3^ ^–^ ^5^ ^)^ the desired target volumes and/or normal tissue volumes encroach into surface buildup dose regions of the patient where the radiation dose from entering photon beams has not reached electronic equilibrium. Buildup doses near the surface are subject to variations in scattered radiation in the form of electrons and photons streaming from the treatment head and thus are dependent of the treatment machine configuration. Under these circumstances, dose to the buildup regions must be accurately calculated by the treatment planning system to yield a valid optimized treatment plan.

There have been many reports on buildup region dose.^(^ ^6^ ^–^ ^11^ ^)^ It is known that the largest contribution to the dose in the buildup region near the surface is from electron contamination emanating from the accelerator head. A lesser contributor is due to low‐energy scattered photons from the head. Electron contamination contributes to the dose at zero depth but decreases rapidly with depth. The low‐energy head‐scattered photons provide a small‐to‐negligible contribution near the surface, but quickly increase their contribution to the dose with depth. Head‐scattered photons have a lower average energy compared to the primary photon field, contributing significantly at shallower depths relative to primary beam photons. Therapy field accessories such as trays and wedges^(^ ^12^ ^–^ ^14^ ^)^ and oblique incidence of the beam^(15)^ also affect the surface dose. Lee et al.^(5)^ reported surface buildup dose measurements for IMRT that included effects of the patient mask material and oblique incidence. They attributed an observed increase in skin toxicity to increased use of oblique angles, increased bolus effect due to the mask material, and inclusion of superficial regions in the target volumes. Dogan and Glasgow^(16)^ reported a decreased buildup region dose when comparing IMRT strip fields to open beam fields. They concluded that any increase in skin toxicity was not inherently due to IMRT delivery techniques.

For megavoltage photon beams using static fields and clinically relevant source‐to‐surface distances (SSD), the contributors to electron contamination are primarily the flattening filter, secondarily the air between the treatment head and patient, and finally the collimation system of the accelerator head.^(6)^ ^,^ ^(7)^ ^,^ ^(17)^ Head‐scattered photons emanate primarily from sources upstream from the secondary collimators (flattening filter and primary collimator).^(18)^ For any point in the measurement plane, the surface‐contaminating radiation dose is primarily a function of the amount of exposed flattening filter viewable through the collimators.^(11)^ Although forward peaked, the spectrum of contaminating electrons has a finite angular spread.^(7)^ The result is a Gaussian‐type distribution of dose near the surface, falling off away from central axis and extending outside of the open field.^(6)^

Buildup region dose is dependent on the position of the collimator leaves during beam delivery and thus can be influenced by delivery technique. In static multileaf collimator (MLC) mode (step and shoot), the collimators move to a position with the beam off and stop before the beam is delivered, then move to the next position while the beam is off. In dynamic MLC mode (dynamic sliding window), the leaves move during beam delivery. Each leaf pair must complete the irradiation in a predetermined manner to meet the treatment goal, and therefore sweep across the field with a varying gap determined by the desired dose distribution. The amount of flattening filter visible while delivering essentially identical treatments may vary significantly for the static versus dynamic modes with impact on the level of contaminating radiation delivered over the field.

An artifact of static or dynamic MLC delivery is that all treated areas experience a significant time under the leaves. Since the transmission factor for the leaves (~2% of open field for Varian MLCs) is significant compared to desired dosimetry precision, the MLC transmission contribution to the useful beam must be taken into account during treatment planning, translation to leaf sequencing for delivery, and monitor unit calculations. Less efficient delivery (i.e., using more monitor units to create the intensity pattern) of the primary field increases the MLC transmission component and influences the buildup region dose.

The effects of segmentation for IMRT delivery on the dose in the buildup regions were investigated. Measurements of surface buildup dose were performed for simple static and dynamic MLC fields and for an example IMRT field. Due to the small field environment resulting from beam segmentation and the lack of electronic equilibrium in the buildup region, measurements from multiple detectors were used and compared. Measurement results are analyzed in terms of beam delivery efficiency and degree of field segmentation.

## II. METHODS AND MATERIALS

### A. Measurement

A Varian 2100EX linear accelerator with 120‐leaf Millennium MLC was used to provide the 6 MV photon beam for the measurements. All the measurements were performed at 90 cm SSD, and jaws were fixed at a width of 14 cm and a length of 10 cm for the uniform fields and at a width and length of 8 cm for the intensity‐modulated fields.

Parallel plate chambers, a stereotactic diode, a scanning ion chamber, and thermoluminescent dosimeters (TLD) were used to measure dose. The Attix parallel plate chamber was chosen for its good depth resolution (1 mm cavity thickness, 12.7 mm diameter collecting volume, 13.5 mm guard ring width, $4.5\;\text{mg}/{\text{cm}}^{2}$ Kapton entrance window); it also requires little empirical correction (<1% of dose at maximum depth, $D_{\max}$ ^(19)^ ^,^ ^(20)^) due to its guard ring geometry and casing material. A disadvantage is that it is not waterproof, and therefore was used to perform measurements in Solid Water (Gammex RMI Model 457). To allow a measurement comparison between water and Solid Water, a waterproof Exradin P11 parallel plate chamber (2 mm gap, 1 mm polystyrene window thickness, 20 mm collecting diameter, and 7 mm guard ring width) was also used. Rawlinson^(19)^ correction factors were used for both chambers. Corrections ranged up to 2.2% in the buildup region. Both chambers used a custom‐machined cavity for the Solid Water measurements.

A stereotactic diode (IBA Scanditronix Medical AB, Uppsala, Sweden) was used because of its small dimension perpendicular and parallel to the beam (thickness 0.06 mm, sensitive volume diameter 0.6 mm), which is advantageous for point measurements in high‐dose gradient regions. The scanning ion chamber (Welhoffer Model CC13) had a diameter of 6 mm and volume of 0.13 cm^3^.

**Figure 1:**
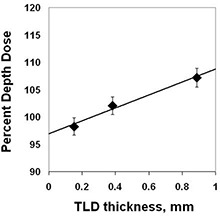
TLD extrapolation method. TLD measurement results for TLD thicknesses indicated are extrapolated to zero thickness. Shown is a measurement for 2 mm depth, $8\times 8\;{\text{cm}}^{2}$ open field.

The TLDs were used because of the small horizontal dimension $\left(3.175\times 3.175\;{\text{mm}}^{2}\right)$ for point dose measurements. In the buildup dose region, it is known that the thickness of the TLD chips causes a systematic error in depth dose measurements. This problem was alleviated by using an extrapolation method (LiF, $3.175\times 3.175\;{\text{mm}}^{2}$ chips with three thicknesses, 0.89 mm, 0.38 mm, and 0.15 mm).^(21)^ The accuracy of this technique was verified using a static open field for comparison of depth doses as measured by the TLD extrapolation method and the Attix chamber. Fig. 1 shows the relative thicknesses of the TLDs and an example extrapolation to zero thickness. A Solid Water holder for the TLD chips that had multiple machined cavities designed to fit the corresponding chip thicknesses was used with the Solid Water stack phantom. All TLDs were calibrated at 10 cm depth for individualized sensitivity factors. Each measurement was repeated three times using three TLD chips of the same thickness. A total of nine measurements were used for the extrapolation. For buildup dose measurements, the TLDs were mounted in Solid Water such that the surface facing the beam was placed at constant depth. A linear extrapolation to zero TLD thickness was performed for each measurement point in the buildup region.

**Figure 2:**
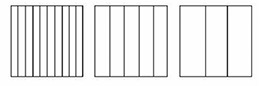
Strip fields. Measurements were performed for the field segments shown. Each sequence summed to a uniform $10\times 10\;{\text{cm}}^{2}$ field.

To study the effects of segmentation and fluence variation of IMRT delivery on the buildup dose, two different types of measurement were performed. Uniform fields of $10\times 10\;{\text{cm}}^{2}$ at the isocenter (100 cm SAD, 90 cm SSD, $14\times 10\;{\text{cm}}^{2}$ jaw setting), with no intensity modulation intended, were delivered as a sum of consecutive rectangular strips. The rectangular strip shape was formed with the MLC as a subfield of the full field. Three combinations of strip widths, 1.0 cm×10,2.0 cm×5, and 3.5 cm+3.0 cm+3.5 cm, all defined at the isocenter, were used for comparison (Fig. 2). The rectangular strips were formed so that the long side (10 cm) was perpendicular to the MLC motion. Dynamic IMRT was also used to deliver a uniform $10\times 10\;{\text{cm}}^{2}$ field using a 1.1 cm leaf gap. A Solid Water stack phantom $\left(40\times 40\;{\text{cm}}^{2}\right)$ was placed in the field. MLC correction^(22)^ values were optimized before the measurement using digitized and dose‐corrected images on Kodak XV‐2 film in Solid Water. The dose profile of the superposition of the adjacent strips along the direction of MLC motion was designed to be as smooth as practical at 10 cm depth compared with a $10\times 10\;{\text{cm}}^{2}$ open field.

The Attix parallel plate chamber, in a custom‐designed Solid Water holder, was placed at the central axis in the phantom. The same number of monitor units was delivered for each strip. Cumulative readings of the electrometer were recorded after each delivery of the strip field. Measurement sets were repeated three times for each polarity of the chamber bias (±300 V) and depth (0.2 cm, 0.5 cm, 1.5 cm, and 10.0 cm). Average readings for each depth were used to minimize the polarity effect and statistical uncertainty (<0.6%, one standard deviation).

For another uniform field test, the stereotactic diode was used to measure the percent depth dose of each 1 cm wide strip contributing to form the $10\times 10\;{\text{cm}}^{2}$ field. A water phantom (Wellhöfer Blue Phantom) was used, and the diode was scanned in the vertical direction slightly off‐axis of the beam (x=0.50 cm and y=0.25 cm, center of the strip adjacent to the central axis) to avoid the strip interface and MLC tongue and groove effects. The same axis was scanned while each of the strips was delivered in turn.

**Figure 3:**
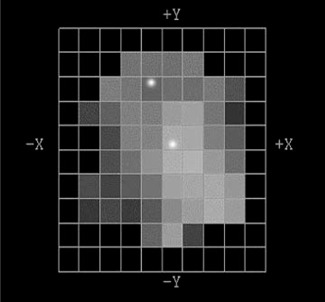
Intensity‐modulated field from a head‐and‐neck treatment plan performed using optimized inverse treatment planning. The beamlet grid pattern is 1 cm^2^ at the isocenter. Bright points indicate measurement locations. Jaws were positioned at $8\times 8\;{\text{cm}}^{2}$.

Intensity‐modulated field tests using the pattern shown in Fig. 3 were sequenced to deliver the same photon dose at 10 cm depth with static and dynamic IMRT delivery techniques. Doses at two points indicated in the figure, one near the central axis and the other off‐axis in the pattern, were measured at multiple depths with TLDs. The horizontal coordinates of the measuring points were adjusted to account for the divergence of the beam. The TLD extrapolation method was applied for the points experiencing a high dose gradient with depth.

Leaf sequencing algorithms were used to define the delivery parameters. For static MLC delivery, leaf sequencing used the Bortfeld algorithm.^(23)^ For dynamic MLC, full leaf synchronization was used.^(24)^ The sequenced pattern was designed to match the intended dose intensity levels to within 1%.

### B. Error analysis

Measurement variations for each parallel plate chamber measurement (average of repeat collection with both polarities) were less than 0.6%. Maximum depth error in setting depths in water was estimated to be 0.1 mm, using a mechanical depth setting device and a careful reference alignment with the water surface. The uncertainty in depth for Solid Water was estimated from central thickness measurements of the plates. Thicknesses were within 3% of nominal (maximum deviation of 0.15 mm).

The TLD readings were assumed to fluctuate according to the normal error distribution (instrument error). Each reading was divided by the individual chip sensitivity factor, determined at the normalization depth of 10 cm. The estimate of error for each TLD measurement was taken from the average standard deviation of the mean of three TLD readings (0.8%). The error in the intercept for the extrapolation of the TLD readings to zero thickness was determined using a linear least‐squares fit. The mean derived error in the intercept value from the extrapolation of the three measurements was 0.9%. The total TLD measurement error (~1.2%) was the propagation of the average TLD reading at the normalization depth (10 cm) and the depth of interest. Only depths shallower than $D_{\max}$ used the extrapolation method. The measurement error estimate at $D_{\max}$ was slightly less (~1.1%). This analysis does not include error due to the uncertainty in depth. However, measurements used identical experimental setups (i.e., same depth of overlying Solid Water), so that the uncertainty in the depth of measurement did not affect the uncertainty estimate for comparison measurements.

## III. RESULTS

**Figure 4:**
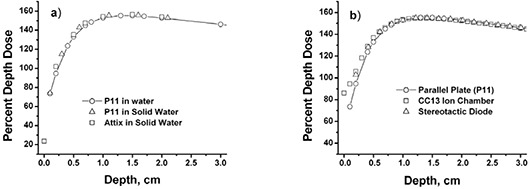
Percent depth dose measurements (a) comparing measurements performed in water and Solid Water using parallel plate chambers; (b) comparing measurements performed with parallel plate chambers, a scanning ion chamber, and a stereotactic diode. Setup geometry was $10\times 10\;{\text{cm}}^{2}$ field size, 90 cm SSD, and 6 MV photons. Depth dose data are normalized to 10 cm depth.

Open field depth‐dose measurements were taken with the detector systems to determine their relative validity as a function of depth. Percent depth dose measurements for the Attix parallel plate chamber and the P11 parallel plate chamber, in water and Solid Water are compared in Fig. 4(a) ($10\times 10{-\text{cm}}^{2}$ open field, 6 MV photons, 90 cm SSD). Measurements were not taken at the same shallow depths because of differing chamber window thicknesses. A systematic difference between the dose measured in water and Solid Water was found. The largest discrepancy (5%) was found at 2 mm depth. Excellent agreement was found elsewhere, particularly at 1 mm depth (P11 polystyrene equivalent window vs. Attix with Solid Water) and depths near $D_{\max}$. Differences were small enough to ignore.

The Attix parallel plate chamber measurements were compared to the TLD extrapolation measurements to test consistency. Good agreement (2.3%, 1.2%, and 1.8% differences at 2 mm, 5 mm, and 15 mm, respectively) was found for an $8\times 8\;{\text{cm}}^{2}$ open field, 90 cm SSD (jaw positions for IMRT field measurements). Differences were consistent with measurement error.

Presented in Fig. 4(b) are measurements taken with the parallel plate chamber, stereotactic diode, and the CC13 scanning ion chamber. All measurements are normalized at 10 cm depth. The CC13 ion chamber and stereotactic diode measurements were in agreement with the parallel plate chamber measurements to within 3% at 4 mm depth and within 2% at 5 mm and deeper depths.

**Figure 5:**
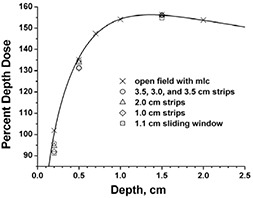
Percent depth dose measurements for uniform $10\times 10\;{\text{cm}}^{2}$ field delivery techniques (6 MV photons, 90 cm SSD). Open field defined by MLC; 3.5 cm, 3.0 cm, 3.5 cm strips; 2.0 cm strips; 1.0 cm strips; 1.1 cm dynamic. Measurements were performed with the Attix parallel plate chamber in Solid Water with $14\times 10\;{\text{cm}}^{2}$ jaw settings and are normalized to 10 cm depth. Solid curve is a fit to the open field measurements.

Buildup dose measurements for the open field, the 3, 5, and 10 strip static delivery, and the dynamic delivery for the $10\times 10\;{\text{cm}}^{2}$ uniform field are shown in Fig. 5. The data are normalized to 10 cm depth for each field. The dose at 2 mm depth decreased 6% (3‐field strip), 7% (5‐field strip), 11% (10‐field strip) and 10% (1.1 cm dynamic) compared to the open field. The buildup dose near the surface is highest for the open field and lowest for the smallest strip field. The dynamic delivery, using an MLC window width of 1.1 cm, produced buildup region dose values similar to the 1 cm segmental strip delivery.

**Figure 6:**
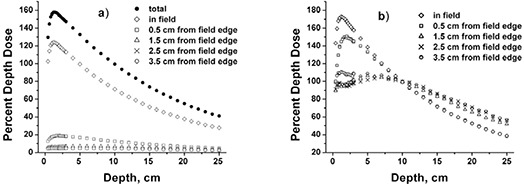
Percent depth dose measurements near central axis (midsegment) for $1\times 10\;{\text{cm}}^{2}$ strip fields. (a) total field and strip field data normalized to total dose at 10 cm depth; (b) strip field data individually normalized to dose at 10 cm depth.

The addition of MLC transmission dose to the treated field has the effect of lowering the buildup region dose because the transmitted radiation is more penetrating (hardened) than the original beam for 6 MV photons.^(25)^ Normalized to a 10 cm depth, contributions at all depths shallower than 10 cm are less than for the open field. The depth dose components measured 0.5 cm off central axis for the 1 cm segmental strip irradiation are presented in Fig. 6. Fig. 6(a) shows the components relative to 10‐cm depth normalization for the total dose, providing the relative contribution for each component. Fig. 6(b) shows the components individually normalized at 10 cm depth. While the individual leaf transmission contributions are relatively small, they add up to ~15% of the total dose at 10 cm for the 1 cm segmental delivery. As can be appreciated from Fig. 6(b), more than 1 cm from the field segment edge, considerably lower dose was received at the shallow depths, relative to the 10 cm depth. The depth doses outside the irradiated segment have contributions from MLC transmission and MLC scattered radiation in addition to in‐phantom scattered radiation from the irradiated segment. The total depth dose is more penetrating with less buildup region dose than the irradiated (in‐field) segment alone.

**Figure 7:**
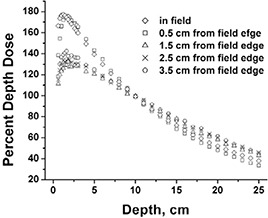
Percent depth dose measurements near central axis for $1\times 1\;{\text{cm}}^{2}$ fields. Data are individually normalized to dose at 10 cm depth.

The contribution of in‐phantom scattered dose slightly decreases the relative buildup region dose because in‐phantom scatter dose peaks at depth relative to that at shallower depths. This effect is shown in Fig. 6(b), where the relative buildup region dose increases with distance from the field edge (see data at near zero depth). To better appreciate this effect, see Fig. 7, which shows data from scans for $1\times 1\;{\text{cm}}^{2}$ fields taken similarly to the scans of Fig. 6. With less in‐phantom scatter, dose due to MLC transmission dominates.

The example intensity‐modulated field, taken from a head‐and‐neck case optimized using inverse treatment planning (Fig. 3), was delivered using static and dynamic IMRT techniques. Point measurements were performed with TLDs both on‐ and off‐axis (Table 1). Measured values near central axis and off‐axis were not significantly different between the two techniques. The off‐axis point doses varied between 40% and 43% of the dose near the central axis for all measured depths (2 mm, 5 mm, 15 mm, and 10 cm). Since the number of monitor units required to deliver the dynamic technique was 21% greater than for the static technique, the dynamic field received a greater contribution from leaf transmission. This effect tended to increase the penetrating ability of the dynamic compared to the static delivery, but was not significant compared to experimental error and was not observed.

**Table 1:** Percent dose measurements (TLD) for the IMRT field using static and dynamic IMRT deliveries. Measurements are normalized to 10 cm depth on the central axis. One standard deviation was estimated at 1.2% for the depth dose measurements and 1.7% for ratio values. Central‐axis and off‐axis measurement locations are shown in Fig. 3.

Depth (mm)		2	5	15	100
static	central axis	98.3	143.6	169.0	100
	off‐axis	41.0	58.0	67.4	41.8
	percent ratio	41.7	40.4	39.9	41.8
dynamic	central axis	97.2	143.6	171.7	100
	off‐axis	40.3	57.2	68.0	42.7
	percent ratio	41.5	39.8	39.6	42.7

**Figure 8:**
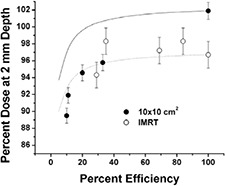
Efficiency of dose delivery versus buildup region dose at a depth of 2 mm. Plotted are data derived from the strip studies for delivery of a uniform $10\times 10\;{\text{cm}}^{2}$ field, for two sequence patterns for an IMRT field near the central axis and off‐axis points, and estimated dose if MLC transmission were the only factor (solid line for $10\times 10\;{\text{cm}}^{2}$ field and dotted line for $8\times 8\;{\text{cm}}^{2}$ field). Doses at 2 mm are relative to the respective doses at 10 cm depth. Measurements were performed in Solid Water. Efficiency was defined as the ratio of the monitor units needed to deliver the unblocked sequence to a point to the total number of monitor units needed to deliver the sequence.

Efficiency of beam delivery at a point was defined as the ratio of the monitor units needed to deliver the portion of the sequence unblocked by the collimators to the total number of monitor units needed to deliver the sequence; the greater the in‐field contribution due to collimator transmission, the less the efficiency. Presented in Fig. 8 is the relationship between the efficiency and the buildup dose at 2 mm depth for the strip tests and the IMRT fields. Off‐axis data are divided by the dose at 10 cm depth plane intersected with the axis of the projected ray line. Data for the $8\times 8\;{\text{cm}}^{2}$ open field (dotted line) and $10\times 10\;{\text{cm}}^{2}$ open field (solid line) are included to show the expected dose if the efficiency of delivery were the only factor. Idealized dose and efficiency for the $10\times 10\;{\text{cm}}^{2}$ and $8\times 8\;{\text{cm}}^{2}$ fields was calculated by varying the contributions to the 2 mm depth dose for the open (jaw‐defined) field and the MLC transmission radiation.

Part of the dependence on the buildup region dose for the segmental $10\times 10\;{\text{cm}}^{2}$ data set is explained by the efficiency analysis. The decline of the dose at 2 mm depth from the 3 cm to the 1 cm strip data (~5%) parallels the efficiency‐only curves. As expected, there is additional reduction of buildup region dose attributable to leaf blocking of the view of the flattening filter (solid line for $10\times 10\;{\text{cm}}^{2}$ data set compared to $10\times 10\;{\text{cm}}^{2}$ strip data). The consistency of the IMRT data with the segmental $10\times 10\;{\text{cm}}^{2}$ data and the efficiency curves suggests a weak dependence (a few percent effect) of the buildup dose on beam delivery efficiency. The equivalent average field size for the IMRT field delivery was approximately $4\times 4\;{\text{cm}}^{2}$ based on MLC aperture analyses.

Compared to a uniform $8\times 8\;{\text{cm}}^{2}$ field represented by the jaw positions, the smaller effective field size reduced the dose at the normalization depth due to a loss of in‐phantom scatter as well as in the buildup region due to a smaller view of the flattening filter.

When the delivery efficiency changes, there is a shift in buildup dose, as illustrated by the strip tests. Mild dependence on efficiency was observed for efficiencies greater than ~20%. The results for the IMRT fields are consistent with this trend. The IMRT field studied did not display unexpectedly large buildup dose dependence, from either decreased view of the flattening filter, delivery efficiency, differences in leaf scatter, or other effects.

## IV. DISCUSSION

For some treatment sites (e.g., head and neck), the target and/or critical structure volumes can encroach into the dose buildup region near the patient's skin. If the treatment planning calculations inadequately estimate the buildup region dose, significant errors can result. The worst case scenario is a loss of control by the treatment planner resulting in a poorly optimized plan. Workaround solutions have included editing volumes to avoid the buildup regions and/or adding bolus to increase confidence in the calculated dose near the skin. Better control over treatment planning strategy would result by validating (possibly improving) the treatment planning calculation algorithm for adequate performance in the buildup region. Given adequate dose estimates, inverse planning algorithms are capable of placing full control of dose levels with the treatment planner, and ultimately with the physician.

Others have reported increased skin toxicity with IMRT for the head and neck.^(5)^ ^,^ ^(16)^ Causes of the increased toxicity have been attributed to increased use of oblique beams, increased bolus effect from mask materials, and including buildup regions in the target volumes. Improvement in treatment planning must include all of these effects. The present work and the work of others^(16)^ have concluded that use of IMRT fields alone decreases buildup dose. An accurate algorithm‐based dose calculation must perform well at perpendicular incidence and oblique incidence and allow for the presence of immobilization materials. The specification of desired dose to target and critical structure volumes, the use of partial bolus, and the optimization of dose are part of the input to the planning process. The present work reports only on perpendicular incidence. Ongoing and planned work will address oblique incidence, immobilization materials, and the interplay between beams to form the full dose distribution.

For IMRT delivery, there was a concern that the contribution from MLC scattered electron contamination could become significant since the edges of the MLC move across the treatment field. However, Nilsson and Brahme^(17)^ concluded that the magnitude of contamination electrons was a function of the angle of primary photon irradiation relative to the angle of collimation. A collimator tipped away from the source produces larger scatter than a collimator tipped toward the source. Thus, the rounded leaf design minimizes collimator scatter. The tongue‐and‐groove edge of the MLC could form a large part of the field edge during IMRT delivery. This potential is minimized by leaf synchronization for dynamic MLC delivery.

Potential differences in buildup region dose between static and dynamic delivery techniques are attributed to (1) the MLC transmission contribution to the useful beam and (2) the contribution associated with contamination originating from the observable part of the flattening filter. For 6 MV photon beams, both effects tend to decrease dose. The contribution from MLC transmission is more profound for less efficient sequencing (i.e., larger monitor units used to deliver the same intensity pattern). The view of the flattening filter from the patient surface is blocked out more effectively using small fields. However, for an MLC mounted downstream from the collimator jaws (as in the Varian 2100 with millennium MLC), an MLC field definition compared to a jaw field definition exposes a greater part of the flattening filter to a point on the patient surface. Thus, even small effective field sizes used for static or dynamic IMRT contribute buildup dose typical of larger jaw fields, minimizing differences in buildup region dose between conformal and IMRT techniques.

The strip tests reported here were designed to investigate the possible extent of the dependence of the buildup region dose on the sequencing technique. The effect was relatively mild (less than 12%) and was shown to be more significant if the dose delivery efficiency was less than approximately 20%. The effect of sequencing on the IMRT fields was small. This result relied on the leaf opening being sufficiently large (i.e., several centimeters) to expose a substantial part of the flattening filter during the open field part of the sequence.

## V. CONCLUSIONS

Buildup region dose deceases for IMRT fields compared to conformal therapy fields for 6 MV, but the general effect is mild (~10%). The dominant characteristics are the addition of dose due to MLC transmission contributing to useful dose while decreasing primary beam exposure and the increase in blocking of the contamination radiation emanating from the treatment head. Since the transmission field is more penetrating for 6 MV and the collimator leaves potentially screen a portion of the contaminating radiation, the surface buildup dose experiences a relative decline.

Of greater concern for treatment toxicity are the added complexities accompanying IMRT, such as oblique fields, increased bolus effect of mask material, and including the buildup region in the target volumes.^(5)^ ^,^ ^(16)^ All of these effects should be included in the treatment planning process. Improved validation of treatment planning algorithms for buildup region dose is needed for perpendicular incidence, oblique incidence, and inclusion of immobilization materials.

## ACKNOWLEDGMENTS

The authors thank Ben Sturm, Nathan Sheets, and Aaron Muncey for help with TLD readout and data analysis.

Supported in part by NIH grant P01‐CA59827.
